# Promotion of High-Speed Copper-Filling Performance for Interconnections with Increasing Aspect-Ratio Using Compound Additives

**DOI:** 10.3390/mi13091539

**Published:** 2022-09-17

**Authors:** Qing Wang, Yang Peng, Yun Mou, Mingxiang Chen

**Affiliations:** 1 School of Mechanical Science and Engineering, Huazhong University of Science and Technology, Wuhan 430074, China; 2 School of Aerospace Engineering, Huazhong University of Science and Technology, Wuhan 430074, China

**Keywords:** through hole filling, high aspect-ratio, defect-free filling, copper interconnection

## Abstract

Interconnections are essential for integrating the packaging substrate, and defect-free copper-filling can further improve the reliability in through holes (THs). The coating properties and filling processes are mainly dominated by the interplays among additives in the direct current electroplating. The acidic copper sulfate electroplating solution contained three typical convection-dependent additives and chloride ions (Cl^−^). The THs with aspect ratios (ARs) of 6.25, 5, and 4.17 (thickness of 500 μm) were selected as the study subjects. The effects of Cl^−^ and ARs on the interactions among the additives were investigated in detail using electrochemical measurements, which were verified by the THs filling experiments. The additive compounds present a convection enhanced inhibition effect and cathodic polarization, leading to a copper filling capacity increase with ARs and the amelioration of copper compactness and corrosion resistance. The defect-free copper filling of THs and a uniform mirror bright surface circuit can be achieved simultaneously using compound additives at a relatively high speed.

## 1. Introduction

Copper is preferred as a good conductor of heat and electricity. Copper electroplating is critical for achieving the interconnection of packaging substrates [1,2,3,4,5]. High-powered devices that work in harsh environments will encounter high temperatures [6,7] and high voltages [8], increasing transmission accuracy [9] and reliability requirements [10]. These excellent performances are based on the properties of the copper layer [11]. The micromorphology, texture, and grain size directly affect the performance of the copper layer and are crucial to device applications. The superb properties are conducive to reducing heat, and improving corrosion resistance and metallurgical properties [12]. The void-free filling of through-holes (THs) can ensure a stable signal transmission [13], and the surface copper lines with uniform thickness are beneficial for reducing or eliminating the grinding process.

Copper electroplating is an efficient method for realizing the interconnections in the packaging substrate, and the copper in the THs and the copper lines on the surface are prepared concurrently. The electrodeposition parameters during the interconnection process have been investigated thoroughly in previous research to control the quality of the copper layer. Among these, the organic additives are the most influential parameter [14], and only a tiny amount is required to adsorb on the cathodic surface to adjust the structure and morphology [15,16]. The electroplating solution contains three additives simultaneously, including a suppressor, accelerator, and leveler. The variety and content of the additives lead to complex adsorption characteristics [17] and multiple interactions as synergistic or antagonistic effects [18,19], which reveals an essential correlation between the copper layer forming process and the additives’ adsorption [20]. Another critical thing to remember is that Cl^−^ plays a pivotal role in electroplating bright copper and improving the stability of additive adsorption [20], and only a tiny amount is needed. It can modify the mechanism of copper electrodeposition and enable ideal properties to occur. In this way, Dow et al. [21] proposed the convection-dependent adsorption (CDA) model, in which the accelerator was verified to have convection-dependent antagonistic effects with inhibition reagents, while convection-dependent synergistic effects are usually presented between the suppressor and leveler [22,23]. Therefore, the effect of additives depends on the adsorption characteristics affected by Cl^−^ concentration and convection, which are critical to increasing the throwing power (TP) to over 100% [24].

The diameter of THs in microelectronic device packaging decrease based on the increasing demand for integration, which blocks mass transfer from the bulk solution to the THs center, and the charges prefer to concentrate at the THs’ mouth [25] during the electroplating process. The above two aspects bring difficulties to the defect-free filling of THs with high ARs. Moreover, with the pursuit of high efficiency, Faraday’s law [26] states that increasing the current density is the most straightforward way to improve the electroplating rate. The charge concentration will be intensified at the THs’ mouth to accelerate the copper electroplating rate [25], and voids are threatened to be created with the closure of the THs’ mouth [27]. The electroplating environment will deteriorate with the current density exceeding the limiting current density of the electroplating solution [28], leading to an enhancement in the copper coating roughness and a performance degradation [29]. Therefore, to fully fill the THs and to electroplate copper lines with high uniformity during one-time electroplating with high speed is an urgent breakthrough direction.

Herein, the additive compounds applicable to high ARs THs copper-filling were obtained. The convection and Cl^−^ on a single additive, and the interactions among the additives were systematically investigated using electrochemical measurements. The optimization effect of Cl^−^ and the additive compounds on the copper coatings’ morphology, corrosion resistance, grain size, and surface preferred orientation were confirmed via the electroplating experiments. The convection-dependent characters were verified via the THs filling experiments with multiple ARs THs. The THs’ filling capability and the surface copper coating leveling capability of the additive compounds were examined.

## 2. Experimental Procedures

### 2.1. Electrochemical Measurements

The main salts in the electroplating solution included 120 g/L CuSO_4_ (AR, Sinopharm, Shanghai, China) and 60 g/L H_2_SO_4_ (AR, Sinopharm). The additives included polyethylene glycol (PEG, molecular weight = 8000, GR, Sinopharm), thiazolinyl polydipropyl sulfonate (SH110, AR, WengJiang, Guangdong, China), and nitrotetrazolium blue chloride (BR, Macklin, Shanghai, China). Cl^−^ was provided by diluting NaCl (AR, Sinopharm) to a specific concentration. Only trace additives were required, and the corresponding concentrations were 200 mg/L, 6 mg/L, and 5 mg/L, respectively. The electrochemical measurement system contains three electrodes, a platinum rotating-disk electrode (RDE) as the working electrode (WE), a platinum sheet with an area of 1 × 1 cm^2^ as the counter electrode (CE), and a mercurous sulfate electrode (MSE) as the reference electrode (RE). The tested solution had a volume of 100 mL. The effect of Cl^−^ on the additives’ adsorption and the copper electrodeposition process was examined via cyclic voltammetric (CV) measurements. The RDE rotates at a speed of 1000 rpm to ensure mass transfer intensification. The Cl^−^ concentration changed from 0 mg/L to 50 mg/L, and the minimum concentration to maintain the stable adsorption of additives was determined by calculating the stripping peak area. The influence of convection on the mechanism of additives during copper electroplating was explored using CV measurements and galvanostatic measurements (GMs). RDE rotation speeds of 10 rpm, 100 rpm, 500 rpm, and 1000 rpm were selected to simulate multiple convection intensities. The CV measurements were implemented within five cycles with a potential range from −0.65 V~1 V vs. MSE, and the current density in GMs was fixed at 2 A/dm^2^.

### 2.2. Copper Filling Experiments

**Figure 1:**
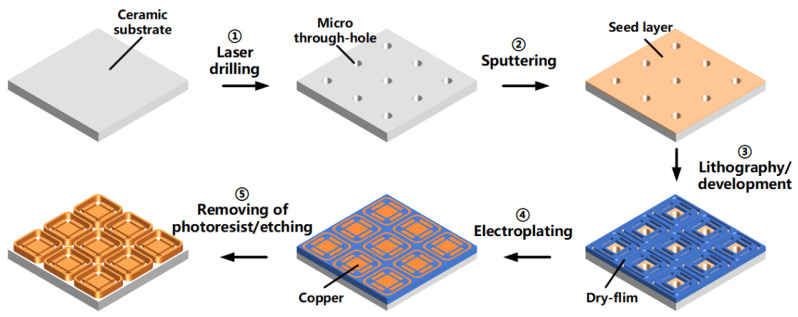
The process of THs filling on the ceramic substrate.

The copper filling experiments were implemented on ceramic substrate, and the process is shown schematically in Figure 1. The THs were prepared via laser drilling. The titanium and copper were then sputtered onto the ceramic surface and the inner wall of the THs successively, to form a seed layer with relative high bonding strength. The dry film was then covered on the seed layer via the heat pressing process, and the parts to be electroplated (THs and circuit) were exposed after photolithography and development. After that, copper was electroplated with a current density of 2 ASD (the same as the GMs). After electroplating, the dry film was removed, and the unnecessary seed layer was etched away.

The effect of each additive and the improvement of copper coating properties using additive compounds with Cl^−^ was examined by observing the THs cross-section and detecting the copper coating properties. The copper filling processes of the THs were obtained using an optical microscope (VHX-6000, KEYENCE, Osaka, Japan), and the throwing power (TP) was also calculated by using the coating thickness ratio at the THs’ center to that near the hole mouth to determine the filling capability. The micromorphologies of the coatings were observed using a field emission scanning electron microscope (FSEM, GeminiSEM300, Zeiss, Oberkochen, Germany). The copper coating texture was explored using an X-ray diffractometer (XRD, Empyrean, PANalytical, Almelo, Netherland), and the grain size and preferred orientation were calculated using the Scherrer Equation and Formula (1), respectively. *I*_(*hkl*)_ and *I*_0(*hkl*)_ are the diffraction intensities of the (hkl) plane measured in the diffractogram for the copper coatings and the standard copper sample. The copper coating corrosion resistance was measured using potentiodynamic polarization curves with 3.5 wt.% NaCl in the three-electrode system. The tested copper coating (1 × 2 cm^2^) is the WE, the saturated calomel electrode (SCE) as the RE, and the Pt sheet (2 × 2 cm^2^) is the CE. The open circuit potential (E_OCP_) was obtained as a function of an exposure time of 0.5 h, and the polarization curves were then recorded from −0.3 V vs. SCE to 0.3 V vs. SCE.

(1) $$TC_{\left(hkl\right)}=\frac{I_{\left(hkl\right)}/I_{0\left(hkl\right)}}{\sum_{i=0}^{n}I_{\left(hkl\right)}/I_{0\left(hkl\right)}}$$

## 3. Results and Discussion

### 3.1. The Effect of Additives on THs Filling

The Cl^−^ concentration significantly impacts on the adsorption characteristics of the additives, which will drastically alter the CV curves by representing a small change. The integrated values of the copper stripping peak area in VMS with the tested additives without or with Cl^−^ were recorded as Q_0_ and Q, respectively. The value of Q/Q_0_ was available to assess the effect of additives and to explore the optimal concentration range of Cl^−^ by determining the functions of the Cl^−^ concentration. CV measurements and analysis were applied to the electroplating solution with a single additive, and the minimum concentration required for the additives to produce a marked effect was determined via linear fitting.

**Figure 2:**
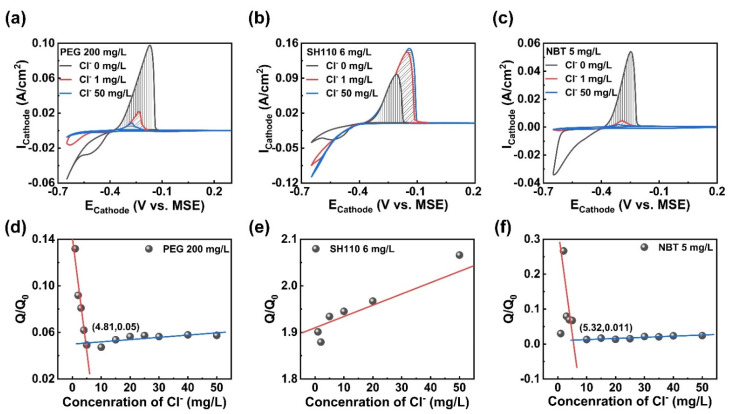
CV measurements of the electroplating solution containing: (**a**) PEG, 200 mg/L; (**b**) SH110, 6 mg/L; (**c**) NBT, 5 mg/L; (**d**–**f**) Q/Q_0_ as a function of Cl^−^ concentration measured in (**a**–**c**).

The CV measurements of the electroplating solution containing a single additive are summarized in Figure 2. Cl^−^ helps the additives to produce a marked effect, and further influences the copper electroplating process. In Figure 2a, the suppressor PEG adsorbs onto the cathodic surface in the molecular state [17,30] without Cl^−^, and the hydrogen evolution occurs during the forward scan (<−0.55 V vs. MSE), with the mass transfer controlled copper electroplating process. In comparison, Cl^−^ enhances the adsorption and suppression effect of PEG; since the values of Q/Q_0_ (Figure 2d) are far below 1, the hydrogen evolution reaction is inhibited, and the starting deposition potential (SDP) and the curves intersect (E_C_, the equilibrium potential of the metallic ion/metal system) [31] in the inset shift negatively. The copper electroplating process converts to kinetics control [32], which helps to improve the surface quality of the copper layer. The PEG achieves a stable adsorption status on the copper cathodic surface, with more than 4.81 mg/L Cl^−^ in the solution.

The CV curves of the accelerator SH110 are depicted in Figure 2b; the acceleration effect of SH110 and the adsorption stability are strengthened via the combination of thiolate and Cl^−^ through facilitation of the electronic delivery process. Firstly, Cl^−^ promotes the nucleation process, since the nucleation loops [32] appear in the reduction process to characterize the formation of a new phase [33], leading to a nucleation change from mass transfer control to kinetics control. Secondly, the copper electrodeposition is accelerated with the steadily rising value of Q/Q_0_ in Figure 2e (the minimum value of 1.8), and the positively moved SDP (from −0.44 V vs. MSE to −0.40 V vs. MSE).

As can be seen in Figure 2c, Cl^−^ significantly increases the adsorption and inhibition effect of the leveler NBT, and the reduction process is also affected. The stripping peak area decreases evidently, and SDP moves in the negative direction (from −0.60 V vs. MSE to −0.64 V vs. MSE); the reduction peak decreases and shifts positively with Cl^−^, and the minimum Cl^−^ concentration meeting the stable adsorption of NBT is 5.32 mg/L. The nucleation processes remain kinetics-controlled. Notably, the inhibiting mechanism in copper electrodeposition is different between PEG and NBT (Figure 2a,c), and the inhibition intensity of PEG (Q/Q_0_ 0.05) is inferior to that of NBT (Q/Q_0_ 0.011).

**Figure 3:**
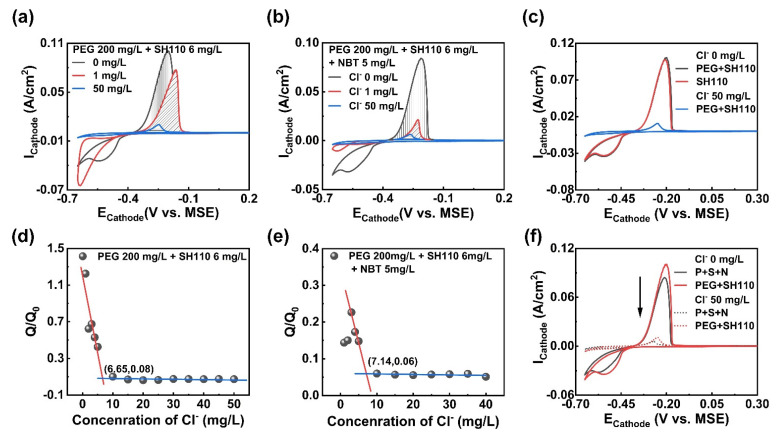
CV measurements of the electroplating solution containing: (**a**) PEG, 200 mg/L + SH110, 6 mg/L; (**b**) PEG, 200 mg/L + SH110, 6 mg/L + NBT, 5 mg/L; (**d**,**e**) Q/Q_0_ as a function of Cl^−^ concentration measured in (**a**,**b**); (**c**,**f**) CV curves comparison for different additives.

The influence of Cl^−^ on the complicated action among multiple additives was explored on the basis of clarifying the interaction between Cl^−^ and a single additive. SH110 and NBT were successively added to the electroplating solution containing PEG, and measured using CV. As shown in Figure 3a, the mixture of PEG and SH110 presents competitive adsorption and antagonistic effects that are affected by Cl^−^ concentration, and the copper electroplating processes are also influenced. The combination of PEG and SH110 first represents the acceleration effect without Cl^−^, and then a suppression effect with increasing Cl^−^ concentration, which can be manifested via the Q/Q_0_ value in Figure 3d, which is much larger than 1 (Cl^−^ 1 mg/L) at the beginning, then rapidly decreases to 0.08 (Cl^−^ 2~5 mg/L), and maintains a strong suppression effect, with Cl^−^ being more than 6.65 mg/L. Moreover, it is much more interesting than Cl^−^ changing the adsorption capability of additives from SH110 > PEG (without Cl^−^) to PEG > SH110 (with Cl^−^). This is because the CV curves of PEG and PEG + SH110 in Figure 3c without Cl^−^ are coincident, and the stripping peak area is much smaller than PEG + SH110 with Cl^−^. The electroplating processes are affected simultaneously, with the “nucleation loop” appearing with a low Cl^−^ concentration.

More intricate interactions exist among the three additives. Firstly, as depicted in Figure 3b, the copper electroplating amount is inhibited with the increasing Cl^−^ concentration. The inhibition effect is further enhanced compared to PEG alone, indicating a synergistic inhibition effect between PEG and NBT by positively charged ion pairs. Secondly, more Cl^−^ is required in order to achieve stable adsorption with the growing amount of additives in Figure 3d,e. Thirdly, antagonistic effects and competitive adsorption appear between SH110 and NBT, since the stripping peak area is slightly smaller than SH110 alone, and NBT can only replace a little SH110. Thirdly, in Figure 3f, the adsorption capability of the additive changes from SH110 > NBT > PEG to PEG > SH110 >NBT, since Cl^−^ promotes the stable adsorption of PEG and makes it become the primary additive to control copper electroplating.

**Figure 4:**
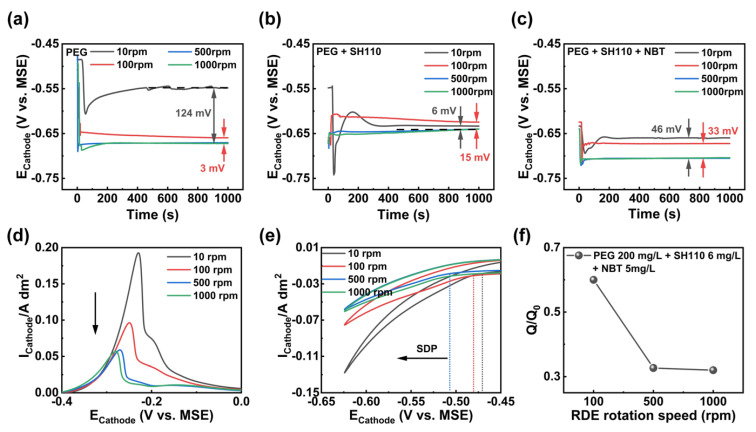
GMs curves (**a**–**c**) and CV curves (**d**–**f**) of the electroplating solution with multiple RDE rotation speeds. (**a**) PEG, 200 mg/L + Cl^−^, 50 mg/L; (**b**) PEG, 200 mg/L + SH110, 6 mg/L + Cl^−^, 50 mg/L; (**c**–**e**) PEG, 200 mg/L + SH110, 6 mg/L + NBT, 5 mg/L + Cl^−^, 50 mg/L; (**f**) Q/Q_0_ as a function of RDE rotation speed.

The additive compound also presents a convection-enhanced inhibition effect with sufficient Cl^−^ in Figure 4. The increasing RDE rotation speed represents strong convection. In Figure 4a, PEG presents a convection-enhanced suppression effect, since the cathodic polarization is enlarged by the increasing convection. With the antagonistic effect between PEG and SH110 in Figure 4b, the cathodic polarization is significantly weakened, except for a RDE rotation speed of 10 rpm. The synergistic effect between PEG and NBT is verified by GM curves in Figure 4c, with the cathodic polarization being enhanced again. Moreover, the stripping peak area decreases sharply (Figure 4d,f), and the starting deposition potential moves towards a more negative potential (Figure 4e) with the RDE rotation speed.

### 3.2. Copper Filling in High ARs THs

**Figure 5:**
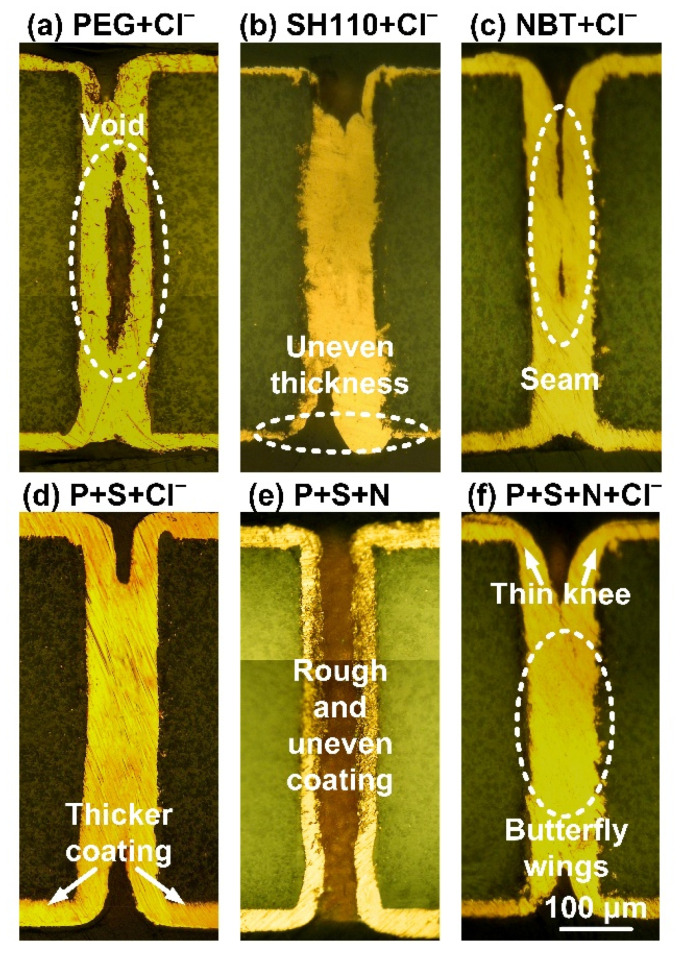
Cross-sections of the THs obtained using electroplating solution with different additives. (**a**) PEG, 200 mg/L + Cl^−^, 50 mg/L; (**b**) SH110, 6 mg/L + Cl^−^, 50 mg/L; (**c**) NBT, 5mg/L + Cl^−^, 50 mg/L; (**d**) PEG, 200 mg/L + SH110, 6 mg/L + Cl^−^, 50 mg/L; (**e**) PEG, 200mg/L + SH110, 6 mg/L + NBT, 5mg/L; (**f**) PEG, 200 mg/L + SH110, 6 mg/L + NBT, 5mg/L, Cl^−^, 50 mg/L.

The additives with sufficient Cl^−^ were applied to the copper filling experiments, and the THs filling capability was examined by observing the cross-sections. The additive compounds promote the defect-free copper filling processes. As can be seen in Figure 5, filling defects are easily formed with a single additive. The convection usually weakens gradually from the THs mouth to the THs center and the convection-dependent adsorption character of the additives compounds, so that the additives will form various distributions in different parts of the THs. A large amount of PEG adsorbs onto the cathode surface, since the molecular weight of PEG is too large to let itself enter the THs. Therefore, the copper electroplating in the THs center is strongly inhibited from forming void (Figure 5a) defects due to the suppressed mass transfer of the cupric ions and the suppression effect of PEG. SH110 presents an acceleration effect with Cl^−^ to significantly accelerate the copper electroplating in the THs center, so that there is a significant copper coating thickness between the center and the surface (Figure 5b). NBT is prone to adsorb on the charge concentration location. The copper electroplating at the THs mouth is inhibited, so more copper can be electroplated at the THs center. The seam is formed in the THs center with the deterioration of mass transfer (Figure 5c).

In contrast, the filling processes of the THs are improved using a combination of additives. In Figure 5d, the coatings in the THs first connect at the center with the competitive adsorption between PEG and SH110. PEG preferentially adsorbs around the THs’ mouth, while SH110 is in the THs’ center. Since SH110 also partially adsorbs onto the surface, the copper coating thickness on the cathodic surface increases simultaneously. Additionally, the THs filling process is further optimized by adding NBT in Figure 5f. The copper coating thickness on the surface slightly decreases, and the coating growth around the THs’ mouth is obviously inhibited, making the copper coatings shaped as butterfly wings. Notably, Cl^−^ is vital to the THs filling process, since the copper coatings appear rough and uneven without butterfly wings (Figure 5e).

**Figure 6:**
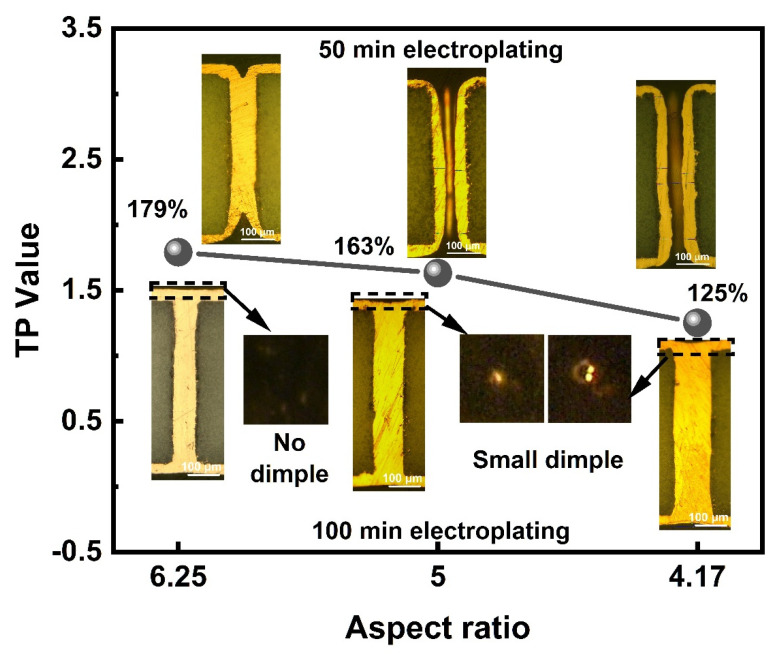
Cross-section and TP values of the defect-free filled THs with high ARs.

**Figure 7:**
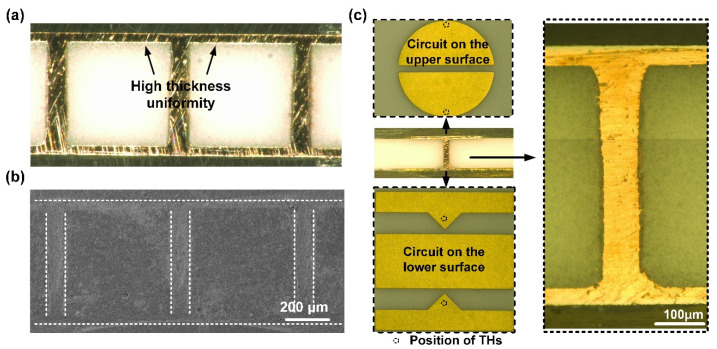
Cross-sections of THs. (**a**) Optical microscope images of THs array, (**b**) SEM images of THs array, and (**c**) optical microscope images of the interconnected substrate.

The THs with high ARs of 6.25, 5, and 4.17 are filled in 50 min to calculate the TP value and 100 min to ensure the closure of the THs mouth. The cross-sections in Figure 6 reveal that the TP increases (from 125% to 179%), and the dimples shrink with the ARs. The electroplating speed is relatively high, since the THs with AR 6.25 are fully filled without a dimple at the mouth. Additionally, the THs with lower ARs can be defect-free filled without a dimple in ample time, the depression is removed in 120 min by the THs with an AR of 5, compared to 140 min by the THs with an AR of 4.17. In Figure 7a,b, the THs array with an AR of 6.25 is filled simultaneously with high surface coating thickness uniformity. The copper electroplating process was applied to the interconnection of the packaging substrate. The circuits on the upper and lower surfaces, and the THs with an AR of 6.25 are fabricated via one-step electroplating, and covered using a Ni/Au thin layer. As shown in Figure 7c, the additive compound is an ideal formula for copper interconnection. The TH is defect-free filled with a uniform surface copper thickness.

### 3.3. Micromorphology, Texture, and Corrosion Resistance

**Figure 8:**
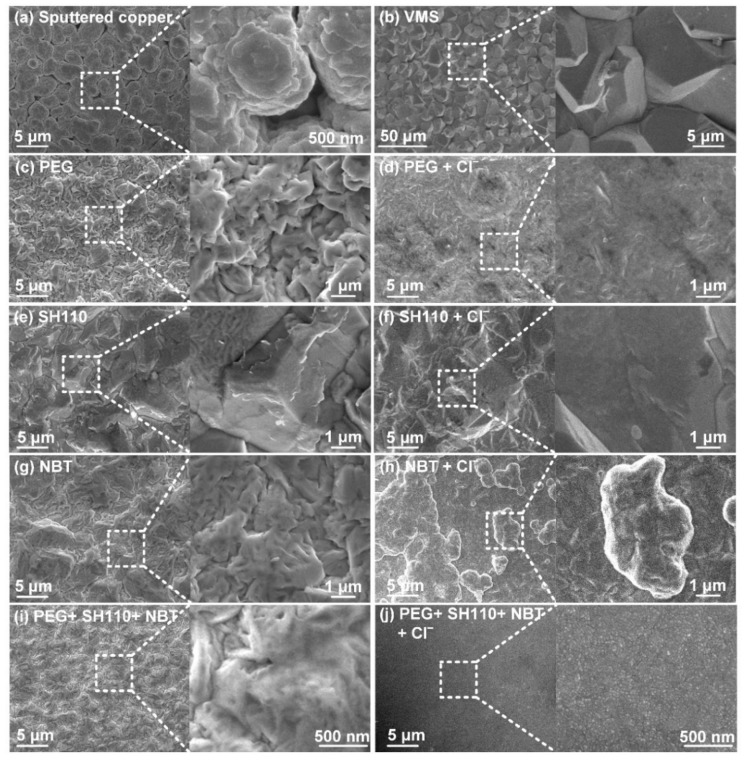
Micromorphology of the copper coatings electroplated on (**a**) sputtered copper, with the electroplating solution containing (**b**) no additive; (**c**) PEG, 200 mg/L; (**d**) PEG, 200 mg/L+ Cl^−^, 50 mg/L; (**e**) SH110, 6 mg/L; (**f**) SH110, 6 mg/L+ Cl^−^, 50 mg/L; (**g**) NBT, 5 mg/L; (**h**) NBT, 5 mg/L + Cl^−^, 50 mg/L; (**i**) PEG, 200mg/L + SH110, 6 mg/L + NBT, 5mg/L; (**j**) PEG, 200mg/L + SH110, 6 mg/L + NBT, 5mg/L Cl^−^, 50 mg/L.

The micromorphologies and roughnesses of the copper coatings vary with the additives [34]. The results are represented in Figure 8. The sputtered copper surface is full of bumps and holes (Figure 8a), and coarse grains are produced without additives (Figure 8b). It can be observed that Cl^−^ improves the Cu surface’s flatness. The surface of SH110 is relatively flat (Figure 8e), while micro-bulges, gullies, and folds appear with PEG (Figure 8c) and NBT (Figure 8g). With the addition of Cl^−^, the surface of PEG is smoothed without micro-bulges (Figure 8d) [35], while SH110 decreases in a concave and convex manner (Figure 8f), and NBT changes from folds to nodulations (Figure 8h). Much more interesting is that the micromorphology uniformity of the additive compounds is the best regardless of Cl^−^, in Figure 8i,j. Especially with the assistance of Cl^−^, the particles are refined and densely arranged, and the macroscopic surface is mirror bright.

**Figure 9:**
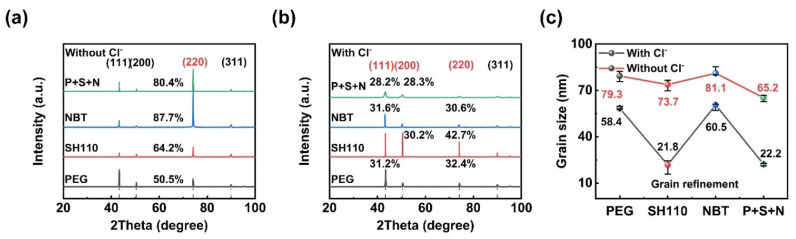
XRD analysis of the electrodeposited Cu layer in the electrolyte: (**a**) with Cl^−^, (**b**) without Cl^−^, and (**c**) grain size and TC.

The influence of Cl^−^ on the preferred orientation and grain size of the copper coatings are explored in detail in Figure 9. The four typical copper peaks of (111), (200), (220), and (311) revealed that XRD patterns are used in TC calculation. The preferred orientation has TC exceeding 25%. The average grain size of the copper coatings was calculated using Scherrer’s formula after adjusting XRD peak broadening for lattice distortions. It is evident in Figure 9a that the preferred orientation of the copper coating is (220) without Cl^−^, which changes to others with Cl^−^ (Figure 9b), and the grain sizes are also affected (Figure 9c). Since the morphology is correlated to the texture [13], exciting connections can be drawn among the texture, micromorphology (Figure 8), and grain size. Firstly, the preferred orientations and micromorphologies of PEG and NBT are the same regardless of Cl^−^ (Figure 8c,g), and they transform the copper coatings’ preferred orientation to (111) to improve the compactness of the coating with the help of Cl^−^. Secondly, SH110 promotes grain refinement and texture adjustment. The grain sizes are much smaller, and the preferred orientation of the (200) peak is only observed in the electroplating solution containing SH110 and Cl^−^. Thirdly, significant grain refinement appears with Cl^−^, and the grain size and preferred orientation are controlled by the three additives at the same time. Therefore, the compact copper coating with a smaller grain size is obtained via the additives compound with Cl^−^.

**Figure 10:**
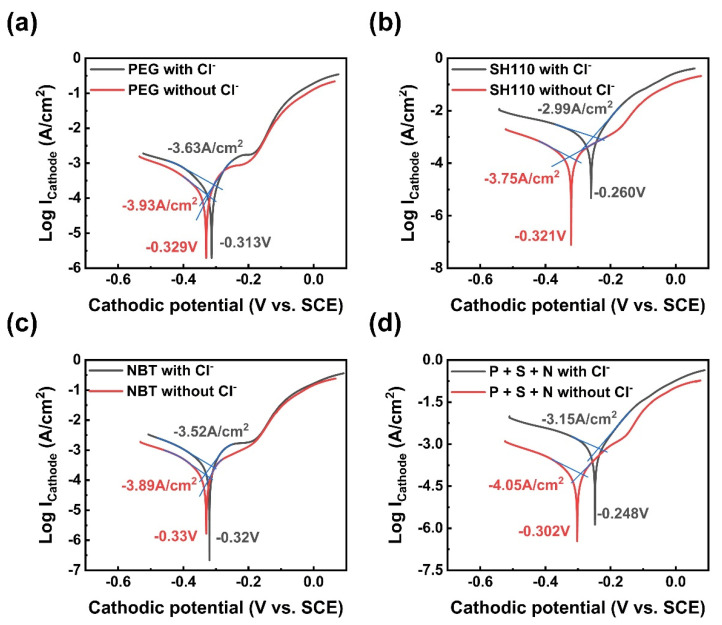
Potentiodynamic polarization curves measured in 3.5 wt.% NaCl solution for the copper coatings. (**a**) PEG, 200 mg/L; (**b**) SH110, 6 mg/L; (**c**) NBT, 5 mg/L; (**d**) PEG, 200 mg/L + SH110, 6 mg/L + NBT, 5 mg/L.

The potentiodynamic polarization curves of the copper coatings electroplated with different additives measured in 3.5 wt.% NaCl solution are shown in Figure 10. The corrosion potential and the corrosion current density are given. It can be seen that a better corrosion resistance can be obtained with Cl^−^ due to its lower corrosion current density. As expected, the copper coating’s corrosion resistance is promoted by the three additives because SH110 promotes the corrosion resistance of the copper coating (Figure 10b,d), which possesses an excellent anti-corrosion capability for the circuit.

As illustrated above, the interactions among the compound additives are conducive to reducing the micro surface roughness, improving the copper coating uniformity and compactness, and promoting grain refinement and corrosion resistance. Therefore, it can be concluded that the copper coating promoted by compound additives can raise reliability in practical applications.

## 4. Conclusions

The defect-free copper filling of THs with excellent coating properties is of great importance for maintaining the reliability of the high integration packaging. This work proposed an ideal convection-dependent additives compound to fill high ARs THs efficiently. The interactions among the additives were strengthened by Cl^−^. The synergistic effect between PEG and NBT inhibited the copper electroplating around the THs mouth with strong convection, while SH110 had an antagonistic effect with the inhibition reagents and promoted copper electroplating at the THs center with low convection, leading to a full fill of high ARs (6.25~4.17) THs with uniform and mirror bright surface copper coatings in 100 min. The filling capability of the additives compound increases with the ARs. The copper coating manifests itself as having excellent corrosion resistance and compactness for work in acidic or salt spray environments. The copper electroplating process has the advantages of low cost and high efficiency; for THs, the surface circuits could be fabricated simultaneously at relatively high speed, and the subsequent grinding processes could be reduced.
